# A Linear-Power-Regulated Wireless Power Transfer Method for Decreasing the Heat Dissipation of Fully Implantable Microsystems

**DOI:** 10.3390/s22228765

**Published:** 2022-11-13

**Authors:** Haochuan Wang, Chenglong Zhu, Wenkai Jin, Junjie Tang, Zhanxiong Wu, Keming Chen, Hui Hong

**Affiliations:** Key Laboratory of Radio Frequency Circuit and System, Hangzhou Dianzi University, Hangzhou 310018, China

**Keywords:** wireless power transfer, linear power regulation, heat dissipation, fully implantable microsystem

## Abstract

Magnetic coupling resonance wireless power transfer can efficiently provide energy to intracranial implants under safety constraints, and is the main way to power fully implantable brain–computer interface systems. However, the existing maximum efficiency tracking wireless power transfer system is aimed at optimizing the overall system efficiency, but the efficiency of the secondary side is not optimized. Moreover, the parameters of the transmitter and the receiver change nonlinearly in the power control process, and the efficiency tracking mainly depends on wireless communication. The heat dissipation caused by the unoptimized receiver efficiency and the wireless communication delay in power control will inevitably affect neural activity and even cause damage, thus affecting the results of neuroscience research. Here, a linear-power-regulated wireless power transfer method is proposed to realize the linear change of the received power regulation and optimize the receiver efficiency, and a miniaturized linear-power-regulated wireless power transfer system is developed. With the received power control, the efficiency of the receiver is increased to more than 80%, which can significantly reduce the heating of fully implantable microsystems. The linear change of the received power regulation makes the reflected impedance in the transmitter change linearly, which will help to reduce the dependence on wireless communication and improve biological safety in received power control applications.

## 1. Introduction

Wireless power transfer (WPT) can enable neural implants with lightweight, miniaturized form factors and sustainable operation, which will benefit the study of the long-term development of neural activity and reveal the working mechanism of the brain [1,2,3]. Options including ultrasonic waves, light illumination, and electromagnetic field are used to deliver energy to implants, but the absorption in skull and tissue limits the power that can be delivered into intracranial implants with ultrasonic or light methods [4,5,6]. Magnetic resonant coupling resonance can wirelessly transmit large amounts of energy robustly with easily implemented structures. Previous studies have shown that wireless power delivery using magnetic resonance coupling can efficiently deliver power to small implantable devices, and is the main method of delivering energy to the implantable brain–computer interface (BCI) [7,8].

Electromagnetic fields impinging on the human body could lead to the power dissipating in tissue and increasing the temperature. For safety reasons, the Federal Communications Commission (FCC) sets the specific absorption rate (SAR) to less than 1.6 W/kg [9,10]. The optimization of coil structures or the adoption of novel metasurface structures can further reduce the specific absorption rate (SAR) [11,12]. However, the existing magnetic coupling resonant wireless power systems are generally optimized for the overall system efficiency, but the efficiency of the wireless power receiving part is not accurately analyzed or optimized [13,14,15,16]. The voltage converter at the receiver may cause additional heat dissipation in the living body, resulting in increased temperature at the implant site. In addition, the impact of long-term and accumulated energy (heat) released on brain activity is difficult to estimate, which may lead to wrong conclusions when using the wireless power fully implantable brain–computer interfaces for the long-term monitoring of biological brain activity [17]. As the heat generated is relatively small, the resulting temperature changes become difficult to measure. Therefore, it is necessary to accurately analyze the structure of the receiver system in wireless power transfer and its influence on heat dissipation, to minimize the heat dissipation of the fully implantable BCI device in the living organism.

**Figure 1:**
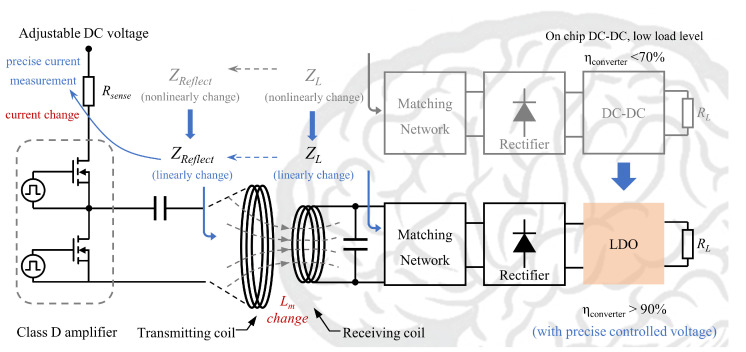
Concept of the linear-power-regulated wireless power transfer system.

Bioimplants such as brain–computer interfaces (BCI) usually have low power consumptions of several milliwatts, so their impedance is typically in the hundreds or thousands of ohms [18,19,20,21,22]. Therefore, the wireless power transfer system for bioimplants usually adopts the series resonant structure of the transmitter (or primary side) and the parallel resonance structure of the receiver (or secondary side), often referred to as S–SP topology, which will reduce the equivalent impedance of the receiver side and improve the overall efficiency of wireless power transfer [13,23,24,25]. Energy received by the secondary side is rectified, and DC-DC is generally used to adjust the output voltage, which makes the wireless power transfer link have a good robust performance to adapt to the received voltage change caused by the air gap change. However, the efficiency of DC-DC is usually below 70% with low power loads, and on-chip DC-DC requires a large area of inductance [26]. Although a variety of on-chip inductance-free DC-DC has been developed, the efficiency is only about 65% [27,28]. Furthermore, the equivalent load impedance will change nonlinearity with the input voltage when the DC-DC is applied on the secondary side, as shown in Figure 1. In the parallel resonance structure, the large nonlinearity change of the equivalent load will easily cause frequency mismatch and reduce the wireless power transfer efficiency. Moreover, the primary side reflected impedance will change nonlinearity, which, together with the frequency mismatch, increases the difficulty of detecting the wireless power transfer status through the primary side. Therefore, in the option of optimal efficiency tracking wireless power transfer, the secondary side voltage and current need to be obtained through wireless communication, and the optimal efficiency tracking needs to be achieved by gradual approximation [29,30,31]. This will lead to slow feedback of control and will easily cause biological safety problems.

Reduction of the secondary side equivalent load resistance variation caused by the primary side input voltage change can stabilize the state of wireless power transfer and reduce energy loss and heat dissipation. By using the LDO to regulate the secondary side output voltage, the equivalent impedance change can be decreased, and the frequency mismatch of the secondary side can be decreased. The output efficiency of LDO can reach 90% under the condition of a low voltage drop, which can further improve the secondary side efficiency of the wireless power transfer system. With the decreased equivalent impedance change, the voltage gain variation can be decreased, which is beneficial for accurate control of the secondary voltage output. Additionally, LDO is easier to integrate on-chip and does not easily cause EMI problems. For the primary side, the class E amplifier circuit is widely used because of its simple structure and high theoretical efficiency [29]. However, the operating angle of the class E amplifier is affected by the load resistance. When the reflection resistance is changed, it is not easy to judge whether the power consumption change is caused by the reflected impedance change or the operating angle change. Although the class D amplifier requires more devices, the operating angle is independent of the load variation, so the reflected impedance can be calculated from the primary side power variation. It is possible to realize the accurate control of the secondary side voltage output through the primary side.

In this paper, an LDO-based linear-power-regulated wireless power transfer method is proposed. The transfer function of the wireless power transfer system with different typologies was obtained by using the gyroscope theory, and the factors affecting the voltage gain were analyzed and verified by simulation. A miniaturized linear-power-regulated wireless power transfer system was developed. The wireless power transfer system was tested, the parameters were changed and the influence of the system efficiency was analyzed.

## 2. Materials and Methods

### 2.1. Parameter Analysis of the Wireless Power Transfer System

**Figure 2:**
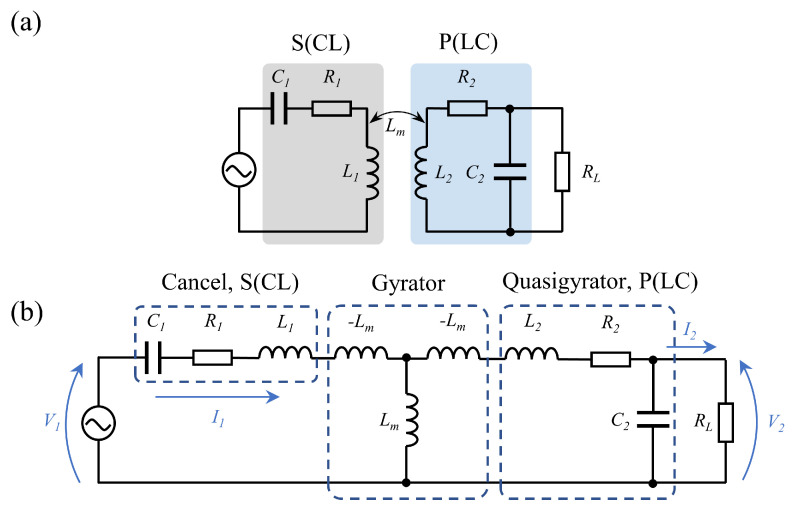
Circuit model of the S−P topology WPT system. (**a**) System structure of S−P topology WPT system, (**b**) equivalent circuit of S−P topology.

Bioimplants are usually implanted with several millimeter-diameter coils; the coupling coefficient between the coils is small so that the optimal load impedance of the wireless power link is usually around a few ohms. To reduce the equivalent load impedance, the primary side in series and the secondary side in parallel (S–P) topology is usually adopted, as shown in Figure 2.

We used the gyroscope theory to calculate the transfer function of S–P topology; $V_{1}$ and $I_{1}$ represent the input voltage and current, $V_{2}$ and $I_{2}$ represent the voltage and current output to the load [32,33,34]. To intuitively show the relationship between input and output and its influencing factors, we did not consider the internal resistances of the transmitter and receiver coil [24,35]. The transfer function is shown as:

(1) $$\begin{array}{cc} \; & \left[\begin{array}{c} V_{1}\\ I_{1} \end{array}\right]=\left[\begin{array}{cc} 0 & -j\omega L_{m}\\ \frac{1}{j\omega L_{m}} & 0 \end{array}\right]\cdot \left[\begin{array}{cc} 0 & j\omega L_{2}\\ j\omega C_{2} & 1 \end{array}\right]\cdot \left[\begin{array}{c} V_{2}\\ I_{2} \end{array}\right]\\ = & \left[\begin{array}{cc} \omega^{2}L_{m}C_{2} & -j\omega L_{m}\\ 0 & \frac{L_{2}}{L_{m}} \end{array}\right]\cdot \left[\begin{array}{c} V_{2}\\ I_{2} \end{array}\right]=\left[\begin{array}{cc} \frac{L_{m}}{L_{2}} & -j\omega L_{m}\\ 0 & \frac{L_{2}}{L_{m}} \end{array}\right]\cdot \left[\begin{array}{c} V_{2}\\ I_{2}, \end{array}\right] \end{array}$$

where $I_{2}=V_{2}/R_{L}$, according to Equation (1), the relationship between output voltage and input voltage can be obtained in Equation (2):

(2) $$\frac{V_{2}}{V_{1}}=\frac{L_{2}R_{L}}{R_{L}\cdot L_{m}-j\omega L_{2}L_{m}}.$$

When the primary side uses a constant voltage source as input, the secondary side can be seen as a constant voltage source output. When the coil parameters are fixed, the output voltage gain is affected by the load resistance $R_{L}$.

Considering the use of an ideal rectifier and voltage regulator, when DC-DC is used to adjust the output voltage, the load can be regarded as a constant power load, thus the equivalent load resistance from the DC-DC input side is $R_{L}=V_{2}^{2}/P_{L}$. Let us consider the influence of input voltage variation on the output voltage. The voltage gain is shown as $V_{2}/V_{1}=g_{s-p}$. When the input voltage changes by *a* times, the input voltage is $a\cdot V_{1}$. Since the input voltage change will cause the voltage gain change, in this case, the voltage gain is expressed as $g_{s-p}^{\prime }$. The variation of secondary voltage is shown in Equation (3), and the variation of receiver equivalent load due to the variation of secondary side voltage $V_{2}$ is shown in Equation (4).

(3) $$a\cdot V_{1}\to g_{s-p}^{\prime }\cdot a\cdot V_{2}$$

(4) $$R_{L}^{\prime }={\left(g_{s-p}^{\prime }\cdot a\right)}^{2}\cdot R_{L}.$$

Substitute Equation (4) into Equation (2) to obtain the changed voltage gain $g_{S-P}^{\prime }$ as shown in Equation (5).

(5) $$g_{s-p}^{\prime }=\frac{\sqrt{4\frac{j\omega L_{m}{}^{2}}{a^{2}R_{L}}+L_{2}{}^{2}}+L_{2}}{2L_{m}}.$$

According to Equations (4) and (5), when the S–P structure has DC-DC for the output voltage regulator, the equivalent load impedance and voltage gain change non-linearly with the primary side voltage and bring two aspects of impact:

1. The variation of the secondary side equivalent load resistance makes the parallel equivalent resistance and the primary side reflected resistance change. The parallel equivalent impedance $Z_{CR}$ is represented by Equation (6), where $R_{CR}$ represents the real part and $jX_{CR}$ represents the imaginary part, as shown in Equations (7) and (8).

(6) $$Z_{CR}=R_{L}//\frac{1}{j\omega C_{2}}=R_{CR}+jX_{CR}$$

(7) $$R_{CR}=\frac{R_{L}}{1+\omega^{2}C_{2}^{2}R_{L}^{2}}$$

(8) $$jX_{CR}=\frac{-j\omega C_{2}R_{L}^{2}}{1+\omega^{2}C_{2}^{2}R_{L}^{2}}.$$

Without considering the internal resistance of the transmitter and receiver coils, the reflected impedance $Z_{refl}$ at the primary side is represented by Equation (9).

(9) $$Z_{refl}=\frac{\omega^{2}L_{m}{}^{2}}{R_{CR}}=\frac{\omega^{2}L_{m}{}^{2}\left(1+\omega^{2}C_{2}{}^{2}R_{L}{}^{2}\right)}{R_{L}}.$$

In the S–P topology, $R_{L}$ changes to $R_{L}^{\prime }$ when the primary side voltage change, the reflection impedance change to $Z_{refl-sp}^{\prime }$, as shown in Equation (10).

(10) $$Z_{ref-sp}{}^{\prime }=\frac{\omega^{2}L_{m}^{2}\left[1+\omega^{2}C_{2}^{2}{\left(g_{s-p}{}^{\prime }a\right)}^{4}R_{L}^{2}\right]}{{\left(g_{s-p}{}^{\prime }a\right)}^{2}R_{L}}.$$

It can be seen from Equation (10) that adjusting the primary side voltage $V_{1}$ causes the nonlinear change of the reflected impedance, which makes the calculation of the secondary side voltage output by the current at the primary side become difficult. In actual use, due to the voltage division caused by the coils and power source, the change of the load impedance will further affect the accurate calculation of the output voltage.

2. The change of the imaginary part causes the off-resonance of the secondary side and reduces efficiency. For parallel resonance on the secondary side, the resonance condition is represented by Equation (11).

(11) $$j\omega L_{2}+jX_{CR}=0.$$

When the primary side voltage changes, the imaginary part of the parallel equivalent impedance is represented by Equation (12).

(12) $$jX_{CR-sp}^{\prime }=\frac{-j\omega C_{2}{\left(g_{s-p}{}^{\prime }a\right)}^{4}R_{L}^{2}}{1+\omega^{2}C_{2}^{2}{\left(g_{s-p}{}^{\prime }a\right)}^{4}R_{L}^{2}}.$$

According to Equation (12), when the load impedance changes greatly, there is a risk of frequency mismatch at the secondary, which affects the voltage gain and reduces the efficiency. $Z_{R}$ represents the residual inductance or residual capacitance caused by the change of load resistance, and the efficiency of the receiver is expressed by Equation (13).

(13) $$\eta_{2-sp}^{\prime }=\frac{R_{CR-sp}^{\prime }}{R_{CR-sp}{}^{\prime }+Z_{R}}.$$

The efficiency of the secondary side is reduced and the reactive power component is increased, so the transmit voltage needs to be further increased to achieve the power consumption required by the load. The increase of the transmitter voltage increases the SAR and endangers biological safety.

**Figure 3:**
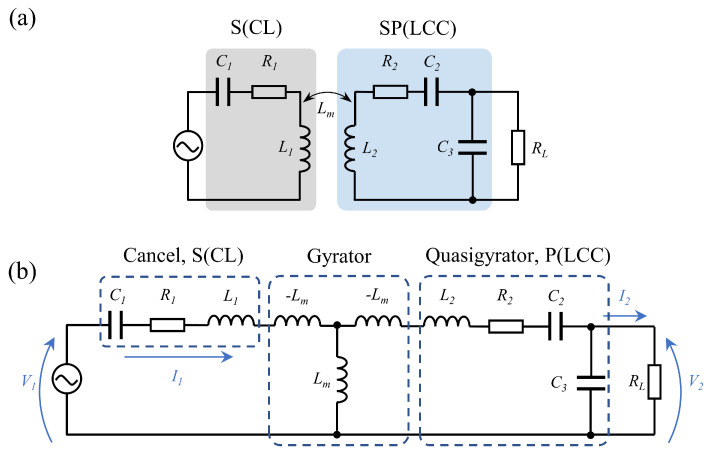
Circuit model of the S−SP topology WPT system. (**a**) System structure of S−SP topology WPT system, (**b**) equivalent circuit of S−SP topology.

The load resistance can be matched through the reactive power matching network, which makes the equivalent load resistance closer to the optimal load impedance [25,36]. The topology formed by adding series capacitors is usually called S–SP topology, as shown in Figure 3.

Similarly, the gyroscope theory was used for analysis, and the S–SP structural transfer function was obtained as shown in Equation (14).

(14) $$\begin{array}{cc} \; & \left[\begin{array}{c} V_{1}\\ I_{1} \end{array}\right]=\left[\begin{array}{cc} 0 & -j\omega L_{m}\\ \frac{1}{j\omega L_{m}} & 0 \end{array}\right]\cdot \left[\begin{array}{cc} 0 & j\omega L_{2}+\frac{1}{j\omega C_{2}}\\ j\omega C_{3} & 1 \end{array}\right]\cdot \left[\begin{array}{c} V_{2}\\ I_{2} \end{array}\right]\\ = & \left[\begin{array}{cc} \omega^{2}L_{m}C_{3} & -j\omega L_{m}\\ 0 & \frac{L_{2}}{L_{m}}-\frac{1}{\omega^{2}C_{2}L_{m}} \end{array}\right]\cdot \left[\begin{array}{c} V_{2}\\ I_{2} \end{array}\right]=\left[\begin{array}{cc} \frac{L_{m}}{L_{2}}\cdot \frac{C_{2}+C_{3}}{C_{2}} & -j\omega L_{m}\\ 0 & \frac{L_{2}}{L_{m}}\cdot \frac{C_{2}}{C_{2}+C_{3}} \end{array}\right]\cdot \left[\begin{array}{c} V_{2}\\ I_{2}. \end{array}\right] \end{array}$$

When using a constant voltage source input, it is similar to the S–P structure and the output has similar constant voltage source characteristics, and $I_{2}=V_{2}/R_{L}$; the voltage gain is given by Equation (15).

(15) $$\frac{V_{2}}{V_{1}}=\frac{L_{2}R_{L}}{R_{L}\cdot L_{m}\left(\frac{C_{2}+C_{3}}{C_{2}}\right)-j\omega L_{2}L_{m}}.$$

$V_{2}/V_{1}=g_{s-sp}$ represents the voltage gain of the S–SP topology. By comparing Equations (2) and (15), it can be seen that with the same coil parameters and load resistance, the voltage gain decreases as $g_{s-sp}<g_{s-p}$ due to the capacitive voltage divider. $\Delta R_{L}=\left|R_{L}-R_{L}^{\prime }\right|$ represents the load variation at the secondary side due to input voltage variation. Compared with S–P topology, S–SP topology brings less load impedance variation, that is, $\Delta R_{L-ssp}<\Delta R_{L-sp}$. Thus the parallel equivalent impedance variation caused by the load impedance variation at the receiver is reduced $\Delta R_{CR-ssp}<\Delta R_{CR-sp}$, the resulting secondary side frequency mismatch is reduced as $\Delta jX_{CR-ssp}<\Delta jX_{CR-sp}$, and the reflected impedance variation is also reduced as $\Delta Z_{refl-ssp}<\Delta Z_{refl-sp}$.

It can be seen from the above analysis that the reduction of the load change makes the wireless power transfer link more stable and the change of the reflected impedance is also reduced.

### 2.2. An LDO-Based Linear-Power-Regulated Wireless Power Transfer Method

**Figure 4:**
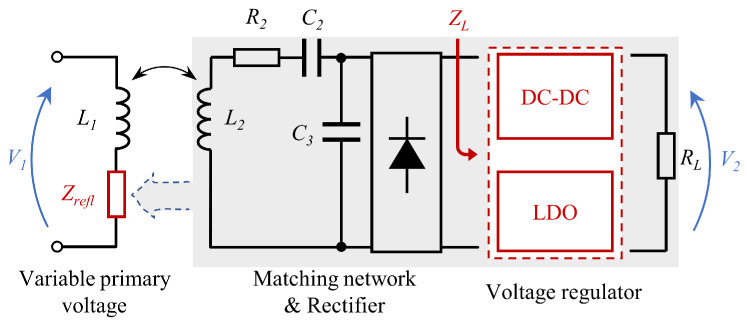
System structure of the secondary side voltage regulator and concept of the reflected impedance in the primary side.

For the further reduction of the load resistance change caused by the primary side input voltage change, and to improve the stability and control accuracy of the wireless power transfer, we considered using LDO as the voltage controller on the secondary side as shown in Figure 4.

The following is a detailed analysis of its impact on wireless power transfer. Since the excess power will be consumed by the LDO, the power consumed by LDO is $P_{LDO}=\left(V_{in}-V_{out}\right)\cdot I_{out}$, when the output voltage is $V_{out}$, the output power is $P_{out}=V_{out}\cdot I_{out}$ and the secondary side load consumes power is $P=P_{LDO}+P_{out}$, so the equivalent load resistance is $R_{L}=V_{in}/I_{out}$. Let the voltage gain as $V_{2}/V_{1}=g$, when the transmitter voltage $V_{1}$ changes, the equivalent load resistance $R_{L}$ change to $R_{L}^{\prime }$, as shown in Equations (16) and (17).

(16) $$a\cdot V_{1}\to g^{\prime }\cdot a\cdot V_{2}$$

(17) $$a\cdot V_{1}\to g^{\prime }\cdot a\cdot V_{2}.$$

Therefore, when LDO is used to adjust the output voltage in the S–SP topology, the resistance change at the secondary side can be represented by Equation (18).

(18) $$\Delta R_{L-LDO}=\left|1-g^{\prime }\cdot a\right|\cdot R_{L}.$$

When DC-DC is used to adjust the output voltage, the resistance change at the secondary side is shown in Equation (19).

(19) $$\Delta R_{L-DCDC}=\left|1-{\left(g^{\prime }\cdot a\right)}^{2}\right|\cdot R_{L}.$$

It can be seen that $\Delta R_{L-LDO}<\Delta R_{L-DCDC}$, and the change of the resistance is linearly related to the change of the primary side voltage. Therefore, it can be known when the input voltage changes, the change of the load resistance is reduced, and the changes of the parameters of the wireless power system such as $\Delta R_{CR}$, $\Delta jX_{CR}$, $\Delta Z_{refl}$, are further reduced. By substituting Equation (17) into Equation (15), the voltage gain can be obtained as Equation (20).

(20) $$g^{\prime }=\frac{L_{2}}{L_{m}}+\frac{j\omega L_{2}}{aR_{L}}.$$

The voltage gain is changed from nonlinear variation to linear variation when LDO is used. The above analysis results show that the use of LDO to adjust the output voltage can further reduce variation of the resonant frequency, the parallel equivalent impedance and the reflection impedance, which provides the possibility for the primary side to precisely control the secondary side output voltage.

**Figure 5:**
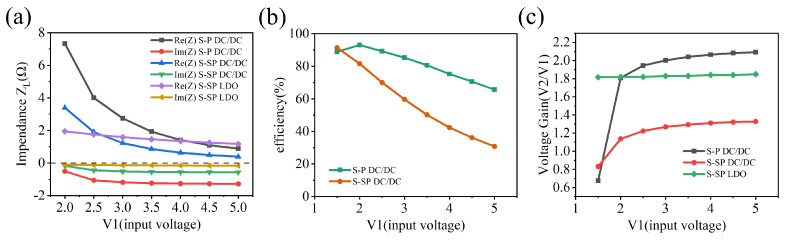
The simulated results of the WPT system. (**a**) The load equivalent impedance varies with the transmit voltage in S−P and S−SP typologies, (**b**) the variation of the WPT system efficiency with transmitting voltage, (**c**) the variation of voltage gain with transmitting voltage.

We performed simulations using Ansys Electronics Desktop Software with an ideal DC-DC and LDO. The load worked at 3.3 V output, 500 Ω load, and approximately 20 mW power. The simulation results are shown in Figure 5. It can be seen that the S–SP topology further reduces the equivalent load resistance in Figure 5a and the state change of the receiver in Figure 5c when the input voltage changes. However, the overall efficiency varies greatly with the input voltage, which is owing to the additional power loss caused by the residual capacitance at the receiver. Using LDO significantly reduces the voltage gain variation compared with using DC-DC, and maintains a relatively stable voltage gain when the input voltage varies greatly.

### 2.3. A Miniaturized Linear-Power-Regulated Wireless Power Transfer System

**Figure 6:**
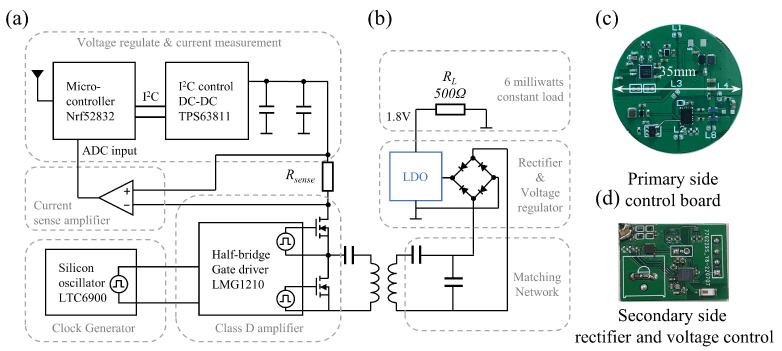
Miniaturized linear-power-regulated wireless power transfer system. (**a**) Schematic of the miniaturized primary side power control system, (**b**) schematic of the LDO-based secondary side wireless power receiver system, (**c**) photograph of the miniaturized primary side power control board, (**d**) photograph of the secondary side wireless power receiver board.

To verify the proposed method of using LDO to realize the parameter of the wireless power transfer system variation linearly, we developed a miniaturized linear-power-regulated wireless power transfer system as shown in Figure 6. The primary side system uses a silicon oscillator (LTC6900, Analog Devices, Inc., Wilmington, MA, USA) to generate a square wave signal with 13.56 MHz frequency, which is supplied to the half-bridge driver (LMG1210, Texas Instruments Inc., Dallas, TX, USA), combined with two GaN MOSFETs (EPC8010, Efficient Power Conversion Corporation, El Segundo, CA, USA) form a Class D amplifier. Because GaN Mosfet has a fast switching speed and the characteristics of low on-resistance, the use of GaN Mosfet has been shown to improve the efficiency of wireless power transfer systems [37]. This Class D amplifier connects to the series resonant capacitor and transmitting coil. The output voltage of the DC-DC (TPS63811, Texas Instruments Inc., Dallas, TX, USA) can be adjusted by the microcontroller (nRF52832, Nordic Semiconductor Inc., Trondheim, Norway) through the $I^{2}C$ bus, which can be adjusted by a 0.2 mV step. Compared with the method of adjusting DC-DC output voltage by DAC, the output voltage regulation is more accurate. The DC-DC output was connected to the input terminal of the Class D Amplifier and used base amplitude modulation to adjust the output voltage of wireless power transfer. A 100 mΩ current sampling resistor was connected in series with the DC-DC output, and a current detection amplifier (INA216, Texas Instruments Inc., Dallas, TX, USA) was used to convert the current value into a voltage value and input it to the internal ADC of the microcontroller to measure the output current.

The secondary side matched the load impedance by adding a series capacitor, and formed the S–SP wireless power transfer topology together with the primary side. The received energy was rectified using a full-bridge rectifier consisting of four Schottky diodes (RB161QS, ROHM Semiconductor, Shanghai, China) with a maximum 0.6 V voltage drop. The rectified voltage was regulated through a low quiescent current 1.8 V output LDO (TPS78218, Texas Instruments Inc., Dallas, TX, USA) and connected to a 500 Ω resistor as a load. The 1.8 V voltage drop usually conforms to the actual working voltage of the miniaturized brain–computer interface chip.

The transmitter coil and receiver coil were simulated by Ansys HFSS. By adjusting the coil size, the coil performance parameters obtained by Ansys HFSS simulation were consistent with those used in wireless power supply simulation. The inductance of the transmitting coil was about 1.5 μH and the resistance was 160 mΩ. The inductance of the receiving coil was 800 nH and the resistance was 100 mΩ. The mutual inductance was about 400 nH in the distance of 5 mm full alignment; the specific parameters are shown in Table 1.

## 3. Results

### 3.1. Measurement of Linear-Power-Regulated Wireless Power Transfer System

**Figure 7:**
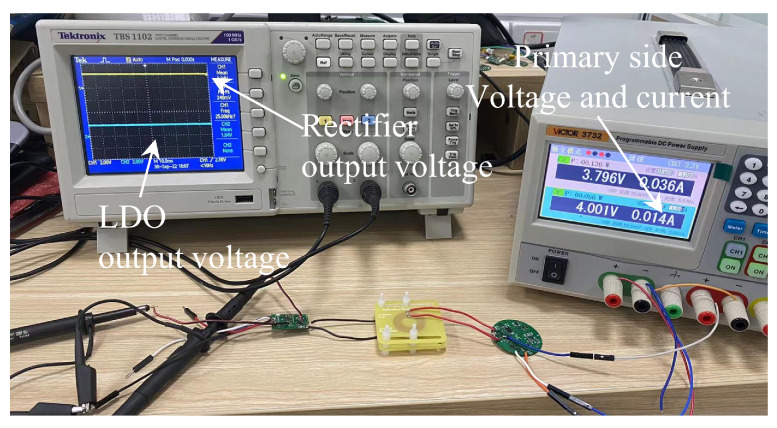
Measurement setup for the linear-power-regulated wireless power transfer system.

The test environment is shown in Figure 7. The oscilloscope was used to measure the rectifier output voltage and LDO output voltage at the secondary side. Because the *I*${}^{2}$*C* control DC-DC in this system can only output a voltage of 1.8–5.5 V, it can only generate a sine wave signal with a maximum amplitude of 5.5/2 × 4/π = 3.5 V with the Class D Amplifier. To expand the measurement range, we used an external voltage source to achieve a wider range of primary side voltage variations. The primary side current is displayed through the voltage source.

**Figure 8:**
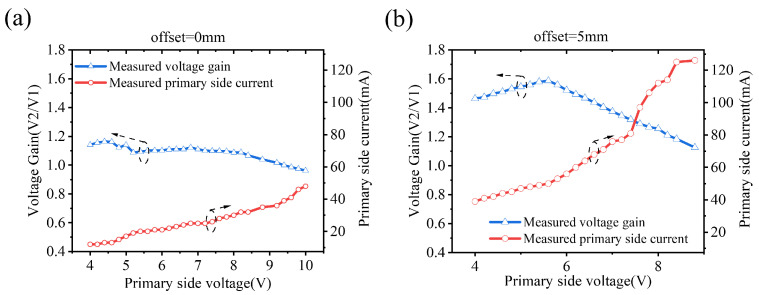
Measurement results of the voltage gain in the power control process and the related primary side current. (**a**) Coils with 5 mm intervals and perfectly aligned, (**b**) Coils with 5 mm intervals and with 5 mm offset.

The test results of voltage gain and primary side current are shown in Figure 8. The input voltage was adjusted by 0.2 V step between 4–10 V, and the output voltage after rectification was measured by oscilloscope. The secondary side measured output voltage value was multiplied by $\sqrt{2}$ to obtain the amplitude of the fundamental wave, and the primary side input voltage $V_{2}$/2 × 4/π to obtain the amplitude of the input fundamental wave. The ratio of output to the input voltage is shown on the left axis of Figure 8a. As the input voltage changes, the voltage gain remains almost constant. However, when the input voltage is high, the voltage gain starts to decrease. There are two reasons for the decrease in voltage gain:

1. As the primary side voltage increases, the power consumption of the circuit increases, which makes the temperature of the circuit increase, therefore resulting in a decrease in voltage gain;

2. When the LDO is used to adjust the output voltage, the current at the secondary side increases synchronously with the primary side voltage, resulting in an increased voltage drop of the rectifier. Make the measured output voltage decrease after rectification.

The current at the transmitter is shown on the right axis. As the input voltage changes, the current linearly increases, indicating that the reflected impedance at the transmitter can be kept stable when LDO is used to adjust the output voltage.

Figure 8b shows the measurement results after the coils are offset by 5 mm. The mutual inductance $L_{m}$ decreases. According to Equations (14) and (15), the voltage gain increases because the ratio of $L_{2}$ to $L_{m}$ increases. As the mutual inductance decreases, the reflected impedance decreases, so the primary side current increases. The voltage gain decline is more serious; this is due to the decreased coupling coefficient makes the primary side need to provide more energy, and the loss in the primary side increases.

### 3.2. Measurement and Analysis of Wireless Power Transfer Efficiency

**Figure 9:**
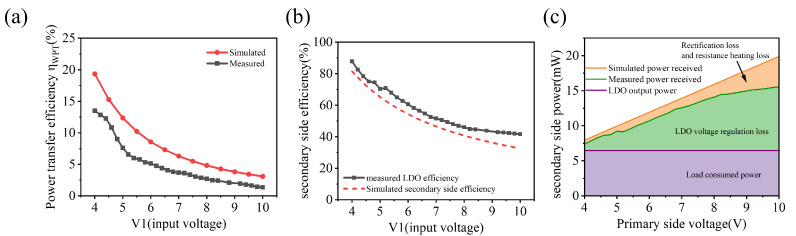
Measurement results of the linear-power-regulated WPT system. (**a**) The power efficiency of the overall WPT system, (**b**) the power efficiency of the secondary side, (**c**) the analysis of the secondary side power consumption.

The overall efficiency of the wireless power transfer system and the partial secondary side efficiency are shown in Figure 9. Figure 9a shows the overall efficiency of the wireless power transfer system. As the input voltage of LDO is much higher than the output voltage when the voltage changes greatly, LDO consumes a large amount of energy, so the efficiency decreases greatly. The actual test results are lower than the simulation results, but keep the same trend as the simulation results.

Figure 9b,c show the efficiency of the secondary side and the analysis of the power consumed. The simulation results show that the receiver efficiency can reach more than 80% when the output voltage after rectification is controlled to be close to the output voltage of LDO. When the LDO input voltage is significantly higher than the LDO output voltage, the loss generated by the LDO and the loss of the rectifier bridge increase synchronously. The voltage drop of the Schottky diode is linearly related to the current flowing through it. Therefore, in the application of low-power implants, the rectifier bridge loss is extremely low. Even if the Schottky diode with the highest voltage drop of 0.6 V is selected, the voltage drop generated by the Schottky diode in the actual test is below 0.3 V. By selecting an ultra-low voltage drop Schottky diode of up to 0.2 V, the bridge loss will be further reduced, allowing the receiver efficiency to reach more than 90%. It is much higher than the receiver efficiency of about 70% when the output voltage is regulated by DC-DC. Therefore, the use of LDO to regulate the output voltage will significantly reduce the heat dissipation in the organism caused by the wireless power transfer, which is conducive to reducing the impact on the neural activity of the organism.

## 4. Discussion

By analyzing the transfer function of the wireless power transfer system, we determined that the load impedance change is the main factor affecting the state of the wireless power transfer when the primary side voltage is changed. In the existing structure, DC-DC is generally used to adjust the output voltage, so that the wireless power transfer system has the strong robust performance to cope with the possible large changes in the coupling coefficient. However, the equivalent load resistance is a non-linear change with the primary side input voltage, causing the nonlinear variation of the resonant frequency and the reflected impedance. The efficiency of on-chip DC-DC is usually less than 70% under low power conditions; the impact of the resulting heating dissipation in the living body is difficult to measure.

When LDO is used to adjust the output voltage, the load impedance change can be from a nonlinear to a linear change and reduces the change amount. When using LDO, it is necessary to strictly control the rectified output voltage to keep the efficiency of the LDO above 90%. Since the change of the reflected impedance is reduced, the change of the coupling coefficient can be calculated and the output voltage can be accurately adjusted. The actual test shows that the efficiency of the secondary side is above 80% when using LDO, and when the rectified output voltage is closer to the LDO output voltage, the efficiency will be further improved. Compared with the use of DC-DC to adjust the output voltage, the use of LDO greatly improves the efficiency of the secondary side, and significantly reduces the heat dissipation in the living body. Moreover, LDO is easier to integrate on-chip, and its small area and low noise features are more conducive to applications such as neural recording. The comparison of on-chip LDO and DC-DC in wireless power transfer is shown in Table 2.

As far as the rectifier loss is concerned, when using DC-DC as a power regulator, the rectifier bridge loss can be reduced by increasing the voltage output. Since the voltage drop caused by the rectifier changes linearly with the flowing current, in the application of implanted devices, due to the low current, the actual test found that the loss caused by the rectifier bridge is extremely low, so it is not particularly important to optimize the efficiency of the rectifier. The selection of ultra-low drop Schottky diodes, semi-active rectifier circuits, or voltage doubler rectifier circuits will further reduce the loss of the rectifier.

In recent years, distributed multi-node brain–computer interface implants have emerged [41,42]. Compared with the use of DC-DC, LDO is more limited in these applications, as the energy transmitted to each implant needs to be independently controlled to maintain the high efficiency of the secondary side. A novel wireless power transfer metasurface will possibly solve this problem [12,43,44,45]. By detecting the reflection resistance generated by each implant and automatically distributing the power transmitted to each implant, the overall efficiency of the wireless power transfer system can be improved while maintaining the high efficiency of each implant.

## 5. Conclusions

We propose a linear-power-regulated wireless power transfer method for decreasing the heat dissipation of fully implantable microsystems in this paper. With the LDO on the secondary side, the parameters of the wireless power transfer system change linearly in the power control process. An LDO-based linear-power-regulated wireless power transfer system was developed and tested; the secondary side efficiency can reach more than 80% with the primary side voltage control, which is significantly improved compared with the use of DC-DC to regulate the output voltage and can effectively reduce the heat dissipation in the organism. The linear-power-regulated wireless power transfer method can reduce the dependence on wireless communication in optimal efficiency tracking applications and improve biological safety.

## Figures and Tables

**Table 1:** Coils specific parameters. Lm represents the mutual inductance of the two coils.

Coil	Turns	Diameter (mm)	Inductance (nH)	Resistance (mΩ)	Lm (nH)
transmitter	9	10	1500	160	400 (d = 5 mm)
receiver	5	10	800	100	

**Table 2:** The comparison of on-chip DC-DC and LDO as the secondary side voltage regulator.

Type	LDO		DC-DC		
Reference	[38]	[39]	[40]	[28]	[27]
Year	2022	2021	2015	2015	2020
Chip Area (mm${}^{2}$)	0.128	0.49	0.04	1.8	36
CMOS Process (μm)	0.18	0.18	0.18	0.18	0.35
Input voltage (V)	1.2–1.8	3.3–3.6	1.8	6–60	0.3–0.4
Output voltage (V)	1	0.8–3.2	1.0–2.2	1.65, 5	1.6–2
Efficiency	83% (max)	96.5% (max)	30–75%	65% (max)	40–65%

## Data Availability

Not applicable.

## Abbreviations

The following abbreviations are used in this manuscript:

FCC	Federal Communications Commission
SAR	Specific absorption rate
BCI	Brain-computer interfaces
WPT	Wireless power transfer
